# Atorvastatin Reduces the Survival of *Candida albicans*-Infected BALB/c Mice

**DOI:** 10.3389/fmicb.2015.01474

**Published:** 2015-12-22

**Authors:** Elias A. Rahal, Wissam N. Constantin, Nabil Zeidan, Alexander M. Abdelnoor

**Affiliations:** Department of Experimental Pathology, Immunology, and Microbiology, Faculty of Medicine, American University of BeirutBeirut, Lebanon

**Keywords:** atorvastatin, *Candida albicans*, interleukin 4 (IL-4), interferon γ (IFN-γ), Statins

## Abstract

Several antimicrobial and immunosuppressive effects have been attributed to the statins class of antihyperlipidemia drugs. Several studies have also indicated clinical benefits for the use of statins during the management of infections and sepsis. To assess whether the immunosuppressive effects of statins outweigh their antimicrobial effects during a fungal infection BALB/c mice were administered *Candida albicans* via intraperitoneal injection. These mice received either a co-injection of atorvastatin along with the infection, were treated with one injection of atorvastatin per day for 5 days prior to infection, or were infected and then treated with one injection of atorvastatin for 5 days afterward. Groups that received *C. albicans* without being treated with atorvastatin were included as controls along with a group that only received phosphate-buffered saline. Mouse survival was then monitored; additionally, serum IFN-γ and IL-4 levels were determined by enzyme linked immunosorbent assay to assess pro-inflammatory and pro-humoral responses, respectively. Atorvastatin administration was capable of altering mouse survival rate with the lowest rate (11.1%) being observed in the group treated for 5 days prior to infection with atorvastatin compared to mice infected but not treated with atorvastatin (44.4%). IFN-γ and IL-4 levels were depressed in all *C. albicans*-infected groups treated with atorvastatin. The possibility that statin administration may suppress or modulate particular components of the immune system during an infection in man should be further explored in large randomized controlled trials.

## Introduction

Statins are HMG-CoA reductase inhibitors that interfere with cholesterol biosynthesis and are thus extensively used in the treatment of hypercholesterolemia. Statins have various pleiotropic effects that are independent of their serum cholestrol-lowering properties. Some of those reported have included antioxidative, anti-inflammatory and immunomodulatory capabilities (Athyros et al., 2009). Members of our group have also reported an enhanced graft longevity effect for atorvastatin, one of the most commonly used statins, in mice and in human transplant recipients; this is potentially due to atorvastatin’s immunomodulatory properties (El-Haibi et al., 2006; Rahal et al., 2012a,b; Zeidan et al., 2013).

A number of studies have indicated that statins may also have beneficial effects as therapeutic agents in infection and sepsis as well as prophylactic agents that reduce the risk of infection. On the other hand, some studies have shown no beneficial effects or even an increased risk of an unfavorable infection outcome. Moreover, a considerable number of meta-analyses and systematic reviews have examined the literature on statins and infection with rather inconclusive results (Falagas et al., 2008; Gao et al., 2008; Kopterides and Falagas, 2009; Tleyjeh et al., 2009, 2012; Bjorkhem-Bergman et al., 2010; Janda et al., 2010; van den Hoek et al., 2011; Ma et al., 2012; Wan et al., 2014; Wang et al., 2014).

Multiple reports indicate that statins have anti-inflammatory and immunomodulatory properties affecting both Th1 and Th2 immune responses (Leung et al., 2003; McKay et al., 2004; Palaniswamy et al., 2010). Statins were demonstrated to interfere with the proliferation and activation of a myriad of immune cell types (Cutts and Bankhurst, 1990; Chakrabarti and Engleman, 1991; Weber et al., 1995; Katznelson et al., 1998; Rudich et al., 1998). Moreover, the expression of a considerable number of cytokines (Weber et al., 1997; Liu et al., 1999), adhesion molecules and class II human leukocyte antigens (HLA) (Kwak et al., 2000) was shown to be downregulated by statins (Rosenson et al., 1999; Kothe et al., 2000; Romano et al., 2000; Wang et al., 2005). On the other hand, statins appear to decrease the expression of cyclooxygenase-2 (COX-2) (Yasmin et al., 2012) while potentially triggering the expression of the rather anti-inflammatory Heme-oxygenase-1 (HO-1) enzyme (Mira et al., 2008). Furthermore, statins may modulate inflammatory responses by interfering with nuclear factor kappa B (NFkB) (Wang et al., 2005), peroxisome proliferator activated receptors (PPAR) and mitogen-activated protein kinase (MAPK) signaling (Kleemann et al., 2004) among other pathways.

Our group has previously reported a suppression of certain aspects of both the humoral and the cell-mediated branches of immunity in mice challenged with egg albumin upon treatment with atorvastatin (El-Haibi et al., 2006). We detected a suppression of IFN-γ and IL-4 production. Therefore, a potential protective effect of statins against infection, as indicated above, in light of its immunosuppressive properties is at first glance rather counterintuitive. Hence, we investigated the effects of atorvastatin on the immune status and on the survival of mice during a fungal infection. The antifungal properties of statins are rather well-documented; various mechanisms that relate to inhibition of ergosterol and isoprenoid-biosynthesis are thought to result in these antifungal properties. Various statins have been reported to inhibit the growth of *Aspergillus* (Macreadie et al., 2006; Yasmin et al., 2012), Zygomycete (Roze and Linz, 1998), *Cryptococcus* (Chin et al., 1997), and *Candida* species including *Candida albicans* (Chin et al., 1997; Song et al., 2003). Mechanisms behind statin-mediated inhibition of fungal growth probably vary between different species. For example, lovastatin-mediated genetic inhibition of siderophore triacetylfusarinine C (TAFC), required for iron uptake in *Aspergillus fumigatus*, results in attenuated virulence in this organism. This implies that statin treatment might affect virulence of *A. fumigatus* also via inhibition of TAFC biosynthesis and not solely via inhibition of isoprenoide biosynthesis. This mechanism is not expected to play a role in fungal species that rely on non-HMGCoA reductase-dependent siderophores or that do not produce siderophores such as *C. albicans* and *Cryptococcus neoformans* (Yasmin et al., 2012).

We intended to evaluate whether the immunosuppressive effects of atorvastatin outweighed its anti-fungal effects. For this purpose BALB/c mice were infected with *C. albicans*. Mouse survival was then monitored. Additionally, IFN-γ and IL-4 were examined in these mice to assess pro-inflammatory and pro-humoral responses, respectively.

## Materials and Methods

### Atorvastatin and *C. albicans*

Lipitor^®^ (Pfizer Inc., New York, NY, USA) tablets, each containing 10 mg of atorvastatin, were pulverized and suspended in phosphate buffered saline (PBS) (Sigma–Aldrich, St. Louis, MO, USA). The *C. albicans* American Type Culture Collection (ATCC) 14053 strain was used in this study. *In vitro* susceptibility of this strain to atorvastatin was verified using Clinical Laboratory Standards Institute (CLSI) guidelines.

### Mouse Treatments and Survival Monitoring

Mouse studies were approved by the Institutional Animal Care and Use Committee (IACUC) at the American University of Beirut prior to initiation of experiments. Five groups of 4–6 weeks old BALB/c mice, each containing 9 animals, were used to monitor survival upon atorvastatin treatment and infection. One group was infected with *C. albicans* receiving a co-injection of atorvastatin, a second group received one injection of atorvastatin per day for 5 days prior to infection with *C. albicans* and a third group was infected with *C. albicans* then treated with one injection of atorvastatin for 5 days afterward. A group that was not treated with atorvastatin but received a single dose of *C. albicans* was included. In addition a group that received a single injection of PBS on the day of infection was used as a negative control since PBS was used to prepare *C. albicans* and atorvastatin doses. Mice infected with *C. albicans* were injected with 10.8 × 10^7^ colony forming units (CFUs) intraperitoneally; this infectious dose was selected since it was sub-LD50 when injected intraperitoneally into BALB/c mice and allowed for monitoring a variation in death events upon atorvastatin treatment. Atorvastatin injections were also administered intraperitoneally with each injection consisting of 40 mg/Kg, the minimum effective immunomodulatory dose in BALB/c mice as reported in our previous studies (El-Haibi et al., 2006). Atorvastatin, *C. albicans* and PBS injections each consisted of 0.25 ml. Infected mice were then monitored for 18 days. Upon mouse death, the liver, spleen and heart were collected, homogenized and cultured to verify that death was due to systemic infection with *C. albicans*.

### Determination of Mouse Serum IFN-γ and IL-4 Levels

To assess mouse serum IFN-γ and IL-4 levels, a set of mice was treated as described above for survival monitoring; however, three mice per group were sacrificed by cardiac puncture on day 3 post-*C. albicans* infection. This day was selected for cytokine assessment since it occurred 1 day prior to escalation of death events. Three mice from the PBS-treated group were also sacrificed on day 3 post-injection. Sera were pooled per group and analyzed for cytokine levels with Single Analyte ELISArray Kits (Qiagen Inc., Valencia, CA, USA).

### Statistical Analysis

The log-rank (Mantel-Cox) test and Kaplan–Meier survival analysis were conducted using PASW Statistics 18 for Windows. Two-sample *t*-tests were used to assess cytokine level variations with the GraphPad *t*-test calculator. Mouse groups infected with *C. albicans* and treated with atorvastatin were compared to the mouse group infected with *C. albicans* but not treated with atorvastatin. *p*-values less than 0.05 were considered statistically significant.

## Results

### Mouse Survival

**FIGURE 1:**
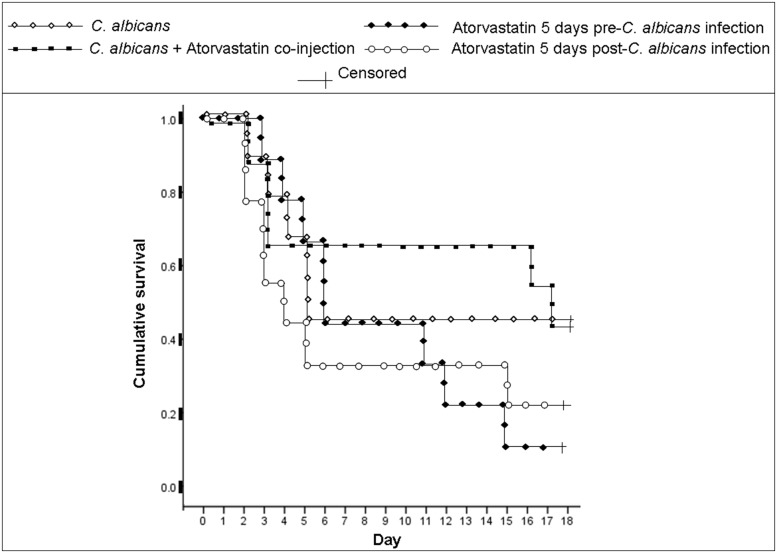
**Cumulative survival of mice infected with *Candida albicans* and treated with atorvastatin.** BALB/c mice received an injection of *C. albicans* and were treated either with a single co-injection of atorvastatin, 5 days pre-infection or a 5 days post-infection atorvastatin regimen. A group infected with *C. albicans* but not treated with atorvastatin was also included. A mouse group injected solely with PBS, as a negative control, showed a 100% survival rate (not shown). Day 0 indicates the day of infection. Mice were monitored for 18 days after infection.

In *C. albicans*-infected mice, survival analysis (*p* < 0.05, individual treated groups compared to the infected but untreated group) revealed that the lowest survival rate at the end of the monitoring period was 11.1%, observed in the group treated for 5 days pre-infection with atorvastatin (**Figure 1**). The survival rate at the end of the monitoring period in the group treated for 5 days post-infection with atorvatstatin was 22.2% while it was 44.4% in the group of mice that received a single dose of atorvastatin along with infection. The survival rate of the group infected with *C. albicans* but not treated with atorvastatin was 44.4%. Mouse death due to *C. albicans* infection was verified via recovering the organism from mouse liver, spleen, and heart by culture; no notable differences in fungal burden were detected between the statin treated groups and the untreated group upon culturing the indicated organs.

#### Mouse Serum IFN-γ and IL-4

**FIGURE 2:**
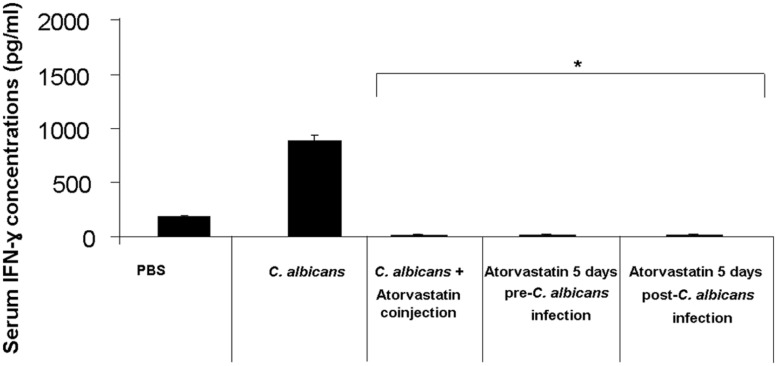
**Serum IFN-γ levels in mice infected with *C. albicans* and treated with atorvastatin.** BALB/c mice received an injection of *C. albicans* and were treated either with a single co-injection of atorvastatin, 5 days pre-infection or a 5 days post-infection atorvastatin regimen. A group that was infected with *C. albicans* but not treated with atorvastatin was included in addition to a group injected solely with PBS, as a negative control. Mouse sera were then assessed for IFN-γ levels by ELISA on day 3 post infection. ^∗^ indicates *p* < 0.05 compared to the *C. albicans*-infected group.

Mouse groups infected with *C. albicans* and treated with atorvastatin showed a 95-fold decrease in the levels of IFN-γ on day 3 post-infection compared to the group infected but not treated with atorvastatin (**Figure 2**).

**FIGURE 3:**
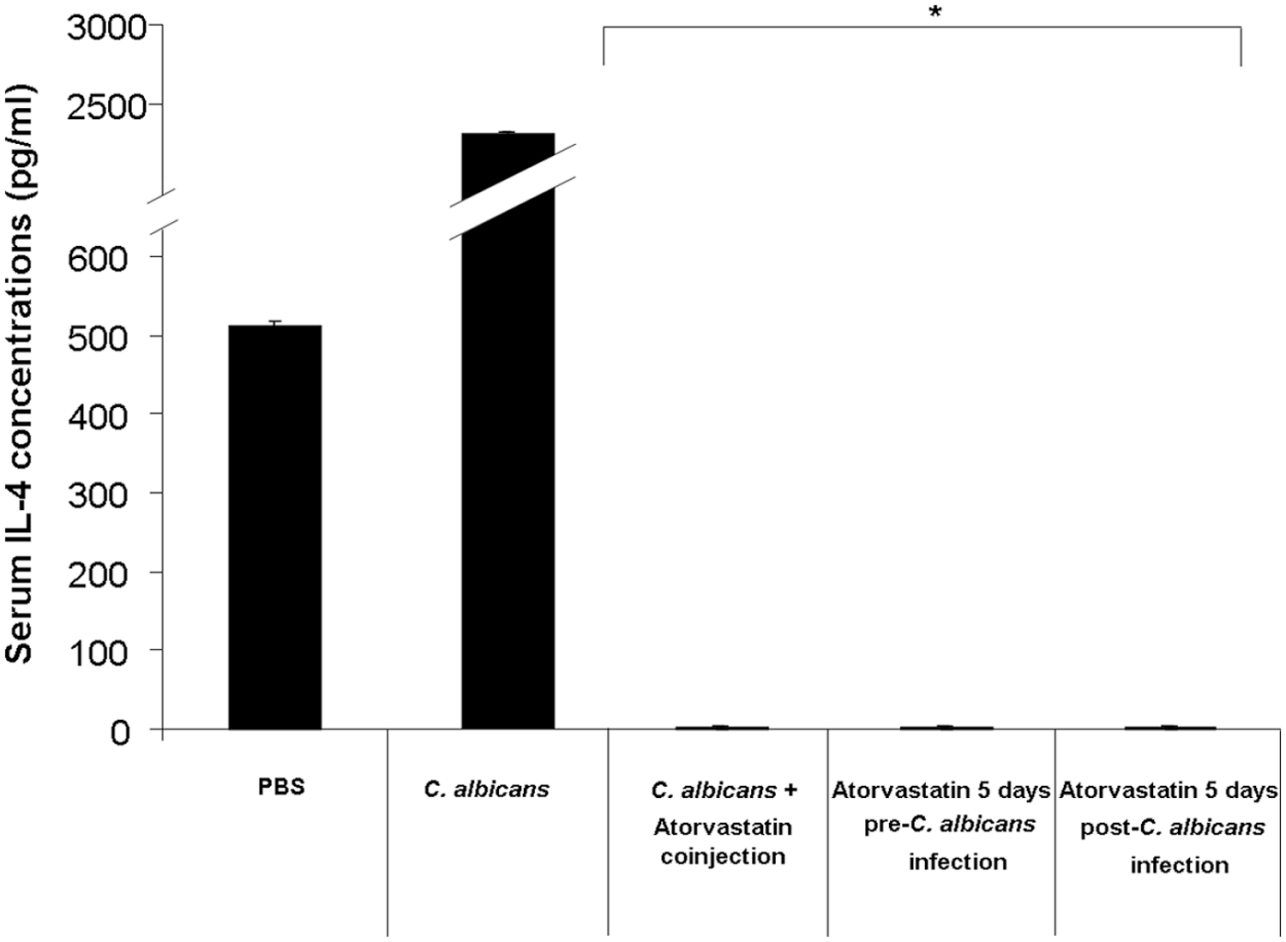
**Serum IL-4 levels in mice infected with *C. albicans* and treated with atorvastatin.** BALB/c mice received an injection of *C. albicans* and were treated either with a single co-injection of atorvastatin, 5 days pre-infection or 5 days post-infection atorvastatin regimen. A group that was infected with *C. albicans* but not treated with atorvastatin was included in addition to a group injected solely with PBS, as a negative control. Mouse sera were then assessed for IL-4 levels by ELISA on day 3 post infection. ^∗^ indicates *p* < 0.05 compared to the *C. albicans*-infected group.

All mouse groups infected with *C. albicans* and treated with atorvastatin showed a suppressed level of serum IL-4 (**Figure 3**). Mice infected with *C. albicans* and treated with atorvastatin displayed a 2222-fold decrease in the level of this mediator on day 3 post-infection compared to mice infected with *C. albicans* but not treated with atorvastatin.

## Discussion

Various antimicrobial and immunomodulatory effects have been described for the statins class of antihyperlipidemia drugs. In addition, several studies have indicated clinical benefits for the use of statins during the management of infections and sepsis in human subjects. We therefore used a mouse model of fungal infection to assess whether the previously described immunosuppressive effects of statins outweigh their anti-microbial effects during an infection. In particular, we examined the effects of atorvastatin, one of the most commonly prescribed statins.

We observed that treatment of *C. albicans*-infected mice with atorvastatin was capable of decreasing survival depending on the regimen employed. IFN-γ and IL-4 levels were suppressed by atorvastatin treatment independent of the treatment mode. We did not detect any notable differences in fungal burden between the statin treated groups and the untreated group upon culturing harvested organs; this may indicate that the immunosuppressive effects detected were sufficient to overcome the antifungal properties of the atorvastatin. Higher doses of atorvastatin may have allowed better tissue distribution and prevented organism proliferation within organ tissues; however, such doses would have been outside of the range used for therapy in humans.

In our previous studies on BALB/c mice atorvastatin reduced the expression of IFN-γ when mice were challenged with albumin (El-Haibi et al., 2006) but not when they received skin allografts from C57BL/6 mice (Zeidan et al., 2013). Hence, a decrease of IFN-γ may be dependent on the challenge itself and whether it can overcome atorvastatin suppression. For example, in a study investigating the *in vitro* role of fluvastatin in *Mycobacterium tuberculosis* infection in human peripheral blood mononuclear cells, fluvastatin was found to enhance the Th1 protective response to the infection (Montero et al., 2000). The proposed mechanism for this effect was an increased activity of Caspase I. This protease converts pro-IL-1β and -18 into their biologically active forms, which in turn act synergistically with IL-12 to induce IFN-γ release; this study found that fluvastatin, in the presence of an *M. tuberculosis* infection, enhances the activity of Caspase I, ultimately leading to an increase in IFN-γ production and therefore giving the immune response a Th1 bias. It has also been shown that statins, including atorvastatin, repress the expression of MHC II molecules induced by IFN-γ, thereby impairing T-cell activation and compromising the adaptive immune response (Kwak et al., 2000). Moreover, simvastatin was shown to suppress the expression of Toll-like receptor (TLR) 4 on human monocytes *in vivo* in response to LPS treatment; this was associated with reduced tumor necrosis factor-α and monocyte chemoattractant protein-1 levels (Niessner et al., 2006). A similar effect was also observed in human CD14+ monocytes treated with atorvastatin, whereby TLR 4 expression was significantly down-regulated (Methe et al., 2005). Hence, upon atorvastatin treatment, whether IFN-γ levels rise or are suppressed in response to a challenge, other aspects of the immune response, including those downstream to IFN-γ, may be impaired. This would result in an inadequate immune response.

All groups treated with atorvastatin had a severe decrease in the levels of the assessed cytokines regardless of the mode of treatment. This corresponded with decreased survival rates in groups of mice treated with atorvastatin for 5 days before or after infection. This indicates that the enhanced death is likely due to the immunosuppressive effects of this agent overcoming its anti-fungal effects. On the other hand, despite the decrease in detection of IFN-γ and IL-4 in the group of mice receiving a single dose of atorvastatin along with infection, death rates were not enhanced in this group. This may indicate that other components of the immune system are affected by a prolonged treatment with atorvastatin resulting in the enhanced death rates seen in the groups of mice treated for 5 days with atorvastatin before or after infection. Components of the immune response previously reported to play major roles in combating *C. albicans* infections that may have been affected by this prolonged treatment include Th17 and neutrophil responses. Multiple studies indicate that these components are modulated by statin treatment. Pravastatin, simvastatin, and atorvastatin were shown to reduce neutrophil transendothelial migration (Maher et al., 2009) and lovastatin was reported to affect leukocyte mobilization and result in a Th1 to Th2 shift in a heat-inactivated *Candida albicans* delayed type hypersensitivity mouse model (Mira et al., 2008). Moreover, simvastatin was demonstrated to enhance neutrophil apoptosis (Chello et al., 2007). Atorvastatin, among other statins, was also shown to decrease Th17 responses in various models and in humans (Aktunc et al., 2011; Li et al., 2011; Jameel et al., 2013; Lappegard et al., 2013). Although the survival rate of the group receiving a single injection of atorvastatin along with infection was similar to the group that received the infection alone by the end of the monitoring period, the death events in the former group occurred at a slower pace. This may indicate that the atorvastatin anti-microbial properties have an effect at this dose that is ultimately insufficient or eventually overwhelmed by the immunosupressed state of the mice.

Worth noting is that the dose of each atorvastatin injection administered to mice in the study at hand, 40 mg/Kg, is equivalent to a dose of 3.24 mg/Kg in man based on United States Food and Drug Administration (FDA) mouse to human dose translation guidelines (Reagan-Shaw et al., 2008). This dose is therefore about 2.5X that currently recommended per day for the treatment of most human subjects. We used this dose since it was the lowest to have an immunomodulatory effect in BALB/c mice in our previous studies (El-Haibi et al., 2006). Moreover, these previous studies have shown that this dose of atorvastatin is not lethal to the mice by itself. While the dose we employed is higher than the one typically employed in man, similar high daily doses have been used in some subject groups (Cilla et al., 1996) and multi-dosing has been reported to result in cumulative blood level spikes (Lins et al., 2003).

A decrease in sepsis-associated mortality rate in human subjects on statins was presumed to be an effect of the immunomodulatory and anti-inflammatory properties of these drugs. In murine models (Merx et al., 2005; Yasuda et al., 2006; Chaudhry et al., 2008; Rosch et al., 2010; Shinozaki et al., 2010), statins appeared to improve animal survival rates after septic shock induced by LPS challenge, bacterial infection or cecal ligation and puncture and pravastatin has been shown to enhance the survival of *C. albicans*- challenged mice (Tashiro et al., 2012). Chaudhry et al. (2008) have demonstrated that mice challenged with LPS have significantly lower serum TNF-α and IL-6, the pro-inflammatory cytokines, upon cerivastatin treatment. On the other hand, animals challenged with live *Staphylococcus aureus* or *Salmonella enterica* serovar Typhimurium did not display a significant decrease in TNF-α or IL-6 levels after treatment.

Although some studies have described potential benefits for statin use during a *Candida* infection both in mice (Tashiro et al., 2012) and in a clinical setting (Forrest et al., 2010; Spanakis et al., 2010; Cuervo et al., 2013), our findings added to the observations denoted above indicate that the effects of statins may be dependent on the type of infection and the agent administered itself in addition to the statin regimen employed. The study by Tashiro et al. (2012), indicated above, reported enhanced survival and a decreased fungal burden in C3H/HeN mice infected with *C. albicans* treated with pravastatin. We did not see a similar effect in BALB/c mice treated with atorvastatin. This underscores the notion that different statins may result in different outcomes potentially in a host and regimen-dependent manner. Further studies will tackle mechanisms upstream of cytokine level modulation that are differently affected in a challenge-dependent manner upon statin treatment. Prominent candidates for examination are members of the TLR family of receptors, some of the first responders that recognize non-host molecules; as described above, evidence indicates that TLR molecules may be affected by statin treatment which rather brings them to the forefront. Moreover, cellular responses downstream of the assessed cytokines resulting in decreased animal survival should also be examined. Therefore, statins are potentially deleterious to the health and survival of a subject during an infection. Their administration may suppress or modulate particular components of the immune system. This possibility should be taken into account and further explored in large randomized controlled trials.

## Conflict of Interest Statement

The authors declare that the research was conducted in the absence of any commercial or financial relationships that could be construed as a potential conflict of interest.

The Reviewer Alhaj-Hussein and handling Editor declared their shared affiliation, and the handling Editor states that the process nevertheless met the standards of a fair and objective review.

## References

[B1] Aktunc E. Kayhan B. Arasli M. Gun B. D. Barut F. (2011). The effect of atorvastatin and its role on systemic cytokine network in treatment of acute experimental colitis. *Immunopharmacol. Immunotoxicol.* 33 667–675. 10.3109/08923973.2011.559475 21428710

[B2] Athyros V. G. Kakafika A. I. Tziomalos K. Karagiannis A. Mikhailidis D. P. (2009). Pleiotropic effects of statins–clinical evidence. *Curr. Pharm. Des.* 15 479–489. 10.2174/138161209787315729 19199976

[B3] Bjorkhem-Bergman L. Bergman P. Andersson J. Lindh J. D. (2010). Statin treatment and mortality in bacterial infections–a systematic review and meta-analysis. *PLoS ONE* 5:e10702 10.1371/journal.pone.0010702 PMC2873291 20502712

[B4] Chakrabarti R. Engleman E. G. (1991). Interrelationships between mevalonate metabolism and the mitogenic signaling pathway in T lymphocyte proliferation. *J. Biol. Chem.* 266 12216–12222. 1712015

[B5] Chaudhry M. Z. Wang J. H. Blankson S. Redmond H. P. (2008). Statin (cerivastatin) protects mice against sepsis-related death via reduced proinflammatory cytokines and enhanced bacterial clearance. *Surg. Infect.* (*Larchmt*) 9 183–194. 10.1089/sur.2006.077 18426351

[B6] Chello M. Anselmi A. Spadaccio C. Patti G. Goffredo C. Di Sciascio G. (2007). Simvastatin increases neutrophil apoptosis and reduces inflammatory reaction after coronary surgery. *Ann. Thorac. Surg.* 83 1374–1380. 10.1016/j.athoracsur.2006.10.065 17383342

[B7] Chin N. X. Weitzman I. Della-Latta P. (1997). In vitro activity of fluvastatin, a cholesterol-lowering agent, and synergy with flucanazole and itraconazole against *Candida* species and *Cryptococcus neoformans*. *Antimicrob. Agents Chemother.* 41 850–852. 9087504 10.1128/aac.41.4.850 PMC163809

[B8] Cilla D. D. Jr. Whitfield L. R. Gibson D. M. Sedman A. J. Posvar E. L. (1996). Multiple-dose pharmacokinetics, pharmacodynamics, and safety of atorvastatin, an inhibitor of HMG-CoA reductase, in healthy subjects. *Clin. Pharmacol. Ther.* 60 687–695. 10.1016/S0009-9236(96)90218-0 8988072

[B9] Cuervo G. Garcia-Vidal C. Nucci M. Puchades F. Fernandez-Ruiz M. Mykietiuk A. (2013). Effect of statin use on outcomes of adults with candidemia. *PLoS ONE* 8:e77317 10.1371/journal.pone.0077317 PMC3796506 24155941

[B10] Cutts J. L. Bankhurst A. D. (1990). Reversal of lovastatin-mediated inhibition of natural killer cell cytotoxicity by interleukin 2. *J. Cell. Physiol.* 145 244–252. 10.1002/jcp.1041450208 2246324

[B11] El-Haibi C. Rahal E. Khauli R. B. Abdelnoor A. M. (2006). Effect of atorvastatin on antibody, interleukin-4 and gamma-interferon production in mice immunized with egg albumin. *Immunopharmacol. Immunotoxicol.* 28 459–470. 10.1080/08923970600928056 16997794

[B12] Falagas M. E. Makris G. C. Matthaiou D. K. Rafailidis P. I. (2008). Statins for infection and sepsis: a systematic review of the clinical evidence. *J. Antimicrob. Chemother.* 61 774–785. 10.1093/jac/dkn019 18263570

[B13] Forrest G. N. Kopack A. M. Perencevich E. N. (2010). Statins in candidemia: clinical outcomes from a matched cohort study. *BMC Infect. Dis.* 10:152 10.1186/1471-2334-10-152 PMC2894022 20525374

[B14] Gao F. Linhartova L. Johnston A. M. Thickett D. R. (2008). Statins and sepsis. *Br. J. Anaesth.* 100 288–298. 10.1093/bja/aem406 18276651

[B15] Jameel A. Ooi K. G. Jeffs N. R. Galatowicz G. Lightman S. L. Calder V. L. (2013). Statin modulation of human T-cell proliferation, IL-1beta and IL-17 production, and IFN-gamma T cell expression: synergy with conventional immunosuppressive agents. *Int. J. Inflam.* 2013 434586 10.1155/2013/434586 PMC3789401 24159421

[B16] Janda S. Young A. Fitzgerald J. M. Etminan M. Swiston J. (2010). The effect of statins on mortality from severe infections and sepsis: a systematic review and meta-analysis. *J. Crit. Care* 25 656.e7–656.e22 10.1016/j.jcrc.2010.02.013 20413251

[B17] Katznelson S. Wang X. M. Chia D. Ozawa M. Zhong H. P. Hirata M. (1998). The inhibitory effects of pravastatin on natural killer cell activity in vivo and on cytotoxic T lymphocyte activity in vitro. *J. Heart Lung Transplant.* 17 335–340. 9588577

[B18] Kleemann R. Verschuren L. De Rooij B. J. Lindeman J. De Maat M. M. Szalai A. J. (2004). Evidence for anti-inflammatory activity of statins and PPARalpha activators in human C-reactive protein transgenic mice in vivo and in cultured human hepatocytes in vitro. *Blood* 103 4188–4194. 10.1182/blood-2003-11-3791 14976045

[B19] Kopterides P. Falagas M. E. (2009). Statins for sepsis: a critical and updated review. *Clin. Microbiol. Infect.* 15 325–334. 10.1111/j.1469-0691.2009.02750.x 19416304

[B20] Kothe H. Dalhoff K. Rupp J. Muller A. Kreuzer J. Maass M. (2000). Hydroxymethylglutaryl coenzyme A reductase inhibitors modify the inflammatory response of human macrophages and endothelial cells infected with *Chlamydia pneumoniae*. *Circulation* 101 1760–1763. 10.1161/01.CIR.101.15.1760 10769273

[B21] Kwak B. Mulhaupt F. Myit S. Mach F. (2000). Statins as a newly recognized type of immunomodulator. *Nat. Med.* 6 1399–1402. 10.1038/82219 11100127

[B22] Lappegard K. T. Pop-Purceleanu M. Van Heerde W. Sexton J. Tendolkar I. Pop G. (2013). Improved neurocognitive functions correlate with reduced inflammatory burden in atrial fibrillation patients treated with intensive cholesterol lowering therapy. *J. Neuroinflammation* 10 78 10.1186/1742-2094-10-78 PMC3699385 23809138

[B23] Leung B. P. Sattar N. Crilly A. Prach M. Mccarey D. W. Payne H. (2003). A novel anti-inflammatory role for simvastatin in inflammatory arthritis. *J. Immunol.* 170 1524–1530. 10.4049/jimmunol.170.3.1524 12538717

[B24] Li X. L. Dou Y. C. Liu Y. Shi C. W. Cao L. L. Zhang X. Q. (2011). Atorvastatin ameliorates experimental autoimmune neuritis by decreased Th1/Th17 cytokines and up-regulated T regulatory cells. *Cell. Immunol.* 271 455–461. 10.1016/j.cellimm.2011.08.015 21889126

[B25] Lins R. L. Matthys K. E. Verpooten G. A. Peeters P. C. Dratwa M. Stolear J. C. (2003). Pharmacokinetics of atorvastatin and its metabolites after single and multiple dosing in hypercholesterolaemic haemodialysis patients. *Nephrol. Dial. Transplant.* 18 967–976. 10.1093/ndt/gfg048 12686673

[B26] Liu L. Moesner P. Kovach N. L. Bailey R. Hamilton A. D. Sebti S. M. (1999). Integrin-dependent leukocyte adhesion involves geranylgeranylated protein(s). *J. Biol. Chem.* 274 33334–33340. 10.1074/jbc.274.47.33334 10559211

[B27] Ma Y. Wen X. Peng J. Lu Y. Guo Z. Lu J. (2012). Systematic review and meta-analysis on the association between outpatient statins use and infectious disease-related mortality. *PLoS ONE* 7:e51548 10.1371/journal.pone.0051548 PMC3524177 23284711

[B28] Macreadie I. G. Johnson G. Schlosser T. Macreadie P. I. (2006). Growth inhibition of *Candida* species and *Aspergillus fumigatus* by statins. *FEMS Microbiol. Lett.* 262 9–13. 10.1111/j.1574-6968.2006.00370.x 16907733

[B29] Maher B. M. Dhonnchu T. N. Burke J. P. Soo A. Wood A. E. Watson R. W. (2009). Statins alter neutrophil migration by modulating cellular Rho activity–a potential mechanism for statins-mediated pleotropic effects? *J. Leukoc. Biol.* 85 186–193. 10.1189/jlb.0608382 18840670

[B30] McKay A. Leung B. P. Mcinnes I. B. Thomson N. C. Liew F. Y. (2004). A novel anti-inflammatory role of simvastatin in a murine model of allergic asthma. *J. Immunol.* 172 2903–2908. 10.4049/jimmunol.172.5.2903 14978092

[B31] Merx M. W. Liehn E. A. Graf J. Van De Sandt A. Schaltenbrand M. Schrader J. (2005). Statin treatment after onset of sepsis in a murine model improves survival. *Circulation* 112 117–124. 10.1161/CIRCULATIONAHA.104.502195 15998696

[B32] Methe H. Kim J. O. Kofler S. Nabauer M. Weis M. (2005). Statins decrease Toll-like receptor 4 expression and downstream signaling in human CD14+ monocytes. *Arterioscler. Thromb. Vasc. Biol.* 25 1439–1445. 10.1161/01.ATV.0000168410.44722.86 15860745

[B33] Mira E. Leon B. Barber D. F. Jimenez-Baranda S. Goya I. Almonacid L. (2008). Statins induce regulatory T cell recruitment via a CCL1 dependent pathway. *J. Immunol.* 181 3524–3534. 10.4049/jimmunol.181.5.3524 18714025

[B34] Montero M. T. Hernandez O. Suarez Y. Matilla J. Ferruelo A. J. Martinez-Botas J. (2000). Hydroxymethylglutaryl-coenzyme A reductase inhibition stimulates caspase-1 activity and Th1-cytokine release in peripheral blood mononuclear cells. *Atherosclerosis* 153 303–313. 10.1016/S0021-9150(00)00417-2 11164419

[B35] Niessner A. Steiner S. Speidl W. S. Pleiner J. Seidinger D. Maurer G. (2006). Simvastatin suppresses endotoxin-induced upregulation of toll-like receptors 4 and 2 in vivo. *Atherosclerosis* 189 408–413. 10.1016/j.atherosclerosis.2005.12.022 16443229

[B36] Palaniswamy C. Selvaraj D. R. Selvaraj T. Sukhija R. (2010). Mechanisms underlying pleiotropic effects of statins. *Am. J. Ther.* 17 75–78. 10.1097/MJT.0b013e31819cdc86 19451808

[B37] Rahal E. A. Chakhtoura M. Dargham R. A. Khauli R. B. Medawar W. Abdelnoor A. M. (2012a). The impact of prophylactic antiviral agents and statin administration on graft longevity in kidney allograft recipients. *Immunopharmacol. Immunotoxicol.* 34 763–767. 10.3109/08923973.2011.653648 22292901

[B38] Rahal E. A. Chakhtoura M. El-Haibi C. Abu Dargham R. Khauli R. B. Medawar W. (2012b). Statins modulate the murine immune response and enhance graft longevity in human kidney transplant recipients. *IOSR J. Pharm.* 2 56–60.

[B39] Reagan-Shaw S. Nihal M. Ahmad N. (2008). Dose translation from animal to human studies revisited. *FASEB J.* 22 659–661. 10.1096/fj.07-9574LSF 17942826

[B40] Romano M. Diomede L. Sironi M. Massimiliano L. Sottocorno M. Polentarutti N. (2000). Inhibition of monocyte chemotactic protein-1 synthesis by statins. *Lab. Invest.* 80 1095–1100. 10.1038/labinvest.3780115 10908155

[B41] Rosch J. W. Boyd A. R. Hinojosa E. Pestina T. Hu Y. Persons D. A. (2010). Statins protect against fulminant pneumococcal infection and cytolysin toxicity in a mouse model of sickle cell disease. *J. Clin. Invest.* 120 627–635. 10.1172/JCI39843 20093777 PMC2810080

[B42] Rosenson R. S. Tangney C. C. Casey L. C. (1999). Inhibition of proinflammatory cytokine production by pravastatin. *Lancet* 353 983–984. 10.1016/S0140-6736(98)05917-0 10459915

[B43] Roze L. V. Linz J. E. (1998). Lovastatin triggers an apoptosis-like cell death process in the fungus Mucor racemosus. *Fungal Genet. Biol.* 25 119–133. 10.1006/fgbi.1998.1093 9974223

[B44] Rudich S. M. Mongini P. K. Perez R. V. Katznelson S. (1998). HMG-CoA reductase inhibitors pravastatin and simvastatin inhibit human B-lymphocyte activation. *Transplant. Proc.* 30 992–995. 10.1016/S0041-1345(98)00123-7 9636401

[B45] Shinozaki S. Inoue Y. Yang W. Fukaya M. Carter E. A. Ming-Yu Y. (2010). Farnesyltransferase inhibitor improved survival following endotoxin challenge in mice. *Biochem. Biophys. Res. Commun.* 391 1459–1464. 10.1016/j.bbrc.2009.12.094 20034462 PMC2813732

[B46] Song J. L. Lyons C. N. Holleman S. Oliver B. G. White T. C. (2003). Antifungal activity of fluconazole in combination with lovastatin and their effects on gene expression in the ergosterol and prenylation pathways in *Candida albicans*. *Med. Mycol.* 41 417–425. 10.1080/1369378031000137233 14653518

[B47] Spanakis E. K. Kourkoumpetis T. K. Livanis G. Peleg A. Y. Mylonakis E. (2010). Statin therapy and decreased incidence of positive *Candida* cultures among patients with type 2 diabetes mellitus undergoing gastrointestinal surgery. *Mayo Clin. Proc.* 85 1073–1079. 10.4065/mcp.2010.0447 21123633 PMC2996154

[B48] Tashiro M. Kimura S. Tateda K. Saga T. Ohno A. Ishii Y. (2012). Pravastatin inhibits farnesol production in *Candida albicans* and improves survival in a mouse model of systemic candidiasis. *Med. Mycol.* 50 353–360. 10.3109/13693786.2011.610037 21954955

[B49] Tleyjeh I. M. Alasmari F. A. Bin Abdulhak A. A. Riaz M. Garbati M. A. Erwin P. J. (2012). Association between preoperative statin therapy and postoperative infectious complications in patients undergoing cardiac surgery: a systematic review and meta-analysis. *Infect. Control Hosp. Epidemiol.* 33 1143–1151. 10.1086/668019 23041814

[B50] Tleyjeh I. M. Kashour T. Hakim F. A. Zimmerman V. A. Erwin P. J. Sutton A. J. (2009). Statins for the prevention and treatment of infections: a systematic review and meta-analysis. *Arch. Intern. Med.* 169 1658–1667. 10.1001/archinternmed.2009.286 19822822

[B51] van den Hoek H. L. Bos W. J. De Boer A. Van De Garde E. M. (2011). Statins and prevention of infections: systematic review and meta-analysis of data from large randomised placebo controlled trials. *BMJ* 343:d7281 10.1136/bmj.d7281 PMC3226140 22127443

[B52] Wan Y. D. Sun T. W. Kan Q. C. Guan F. X. Zhang S. G. (2014). Effect of statin therapy on mortality from infection and sepsis: a meta-analysis of randomized and observational studies. *Crit. Care* 18 R71 10.1186/cc13828 PMC4056771 24725598

[B53] Wang G. Zhang Y. Xie X. Han D. Wu Y. Li S. (2014). [Effect of statins on occurrence of infection and infection-related mortality: a meta-analysis]. *Nan Fang Yi Ke Da Xue Xue Bao* 34 988–993. 25057070

[B54] Wang H. R. Li J. J. Huang C. X. Jiang H. (2005). Fluvastatin inhibits the expression of tumor necrosis factor-alpha and activation of nuclear factor-kappaB in human endothelial cells stimulated by C-reactive protein. *Clin. Chim. Acta* 353 53–60. 10.1016/j.cccn.2004.10.007 15698590

[B55] Weber C. Erl W. Weber K. S. Weber P. C. (1997). HMG-CoA reductase inhibitors decrease CD11b expression and CD11b-dependent adhesion of monocytes to endothelium and reduce increased adhesiveness of monocytes isolated from patients with hypercholesterolemia. *J. Am. Coll. Cardiol.* 30 1212–1217. 10.1016/S0735-1097(97)00324-0 9350917

[B56] Weber C. Erl W. Weber P. C. (1995). Lovastatin induces differentiation of Mono Mac 6 cells. *Cell Biochem. Funct.* 13 273–277. 10.1002/cbf.290130408 8565148

[B57] Yasmin S. Alcazar-Fuoli L. Grundlinger M. Puempel T. Cairns T. Blatzer M. (2012). Mevalonate governs interdependency of ergosterol and siderophore biosyntheses in the fungal pathogen *Aspergillus fumigatus*. *Proc. Natl. Acad. Sci. U.S.A.* 109 E497–E504. 10.1073/pnas.1106399108 22106303 PMC3286978

[B58] Yasuda H. Yuen P. S. Hu X. Zhou H. Star R. A. (2006). Simvastatin improves sepsis-induced mortality and acute kidney injury via renal vascular effects. *Kidney Int.* 69 1535–1542. 10.1038/sj.ki.5000300 16557230 PMC2377392

[B59] Zeidan N. El-Rami F. Al-Akl N. S. Abdelnoor A. M. (2013). The effect of atorvastatin (lipitor) on the duration of survival of allogeneic skin graft and the growth of B16F10 melanoma cells in mice. *Br. J. Med. Med. Res.* 3 1938–1951. 10.9734/BJMMR/2013/3338

